# Topics of Public Interest

**Published:** 1915-02

**Authors:** 


### TOPICS OF PUBLIC INTEREST.

---

**Automobile and Other Highway Mortality.** Automobiles killed, in 1914, 290 persons in New York City; 72 in Buffalo; 24 in Rochester; 16 in Albany; 15 in Syracuse; 8 in Utica; total for the state 600. In 1913, 302 were killed in New York City; 149 for the rest of the state. Trolleys and wagons killed 288 in New York City in 1914; 278 in 1913; total for the rest of the state: 93 in 1913; 111 in 1914. For the whole state, railroads killed 199 persons and destroyed 35 automobiles and 31 wagons in 1914. The total traumatic mortality due to the various kinds of traffic was 1087 which is about 1 per cent. of the total deaths. This 1 per cent. almost entirely preventable and mainly by eliminating that form of egoism which makes each pedestrian or driver feel that others must look out for him.

---

**Hospital Whirlwind Financial Campaigns.** "If your hospital needs money, either to pay its indebtedness or build an addition or a Nurses' Home" etc., says an advertisement. Philanthropy has been a large tax on poor and rich alike in the last few years, irrespective of hard times. Donors have a right to the assurance that every cent contributed, save absolutely inevitable and insignificant expenses, shall go to the cause and that no one is deriving personal benefit from the campaign.

---

**The Axilla,** long known to anatomists as a region of importance and some difficulties, though not having succeeded in enlisting the interest of physiologic chemists in its peculiar aromatic products to the full degree which they deserve, has been taken up by society. The time was when it was considered, not so much immodest as indecent, to display this part of the anatomy. It had the same humble rank—not to imply rankness—as the interdigital spaces of the lower extremities. Until recently, we have been inclined to regard the very frank axillary displays noted after office hours, as due to bad dressmaking, carelessness and accident. Evidence of epilation, depilation or the barber's art, and of deliberate fashioning of gowns leaves no doubt as to intention to force upon the male sex, an appreciation of a hitherto disdained feminine charm.

---

**The "Buy a Bale of Cotton" Fallacy.** The Nash Hardware Co. of Ft. Worth, Texas, has issued a circular beginning "We are not in the cotton business but we know enough to say that an article is only worth what it will bring when offered for sale." It notes the great reduction of expense for various supplies of hardware and farm machinery and holds that while the raiser has a perfect right to hold out for an advanced price, he has not the right to do so by delaying his payments to creditors. "It is hard on the farmer to be disappointed about the price which he thought he was going to get, but it is a whole lot harder on the retail merchant to go broke because the farmer wont sell his cotton and pay his honest debts." It is no secret that nearly all kinds of business have suffered in the last year, not so much from failure of production, as from inability to keep money in circulation. The medical profession has suffered, not only from this cause but from an unaccountably low incidence of disease, not even the charity wards of hospitals having more than a fraction of their usual number of cases. No one hears the medical profession publishing a demand to the public to make an unnecessary call for their services. Our drug manufacturers are not advertising to physicians to prescribe their medicines to cases that do not need them. It is the unquestioned duty for every one who can do so, to buy as liberally as possible to supply his needs. Such a course will help the whole country. But we can see no reason for singling out any special line of industry for special favor to the extent of urging the duty to buy something which the purchaser cannot use in its raw state. Certainly, if any one industry is to be thus favored, it should not be one which will have the balance of demand and supply righted by the effects of the European War, more promptly and more directly than for most businesses.

---

**The Buffalo Depot** of Parke, Davis & Co. has been moved to the 6th floor of the Lincoln Bldg., 323-331 Washington Street.

---

**The Epidemic of Small Pox** on the Tuscarora Reservation, introduced by a visitor from an Ontario Reservation, has been checked by general vaccination. It may be of interest to note that, in the 17th and 18th centuries, a good many small tribes of Indians were almost annihilated by small pox and even by what are usually considered the milder exanthemata. The greater virulence among the aborigines is ascribed to lack of partial hereditary immunity, and to lack of treatment, especially to the fact that, to relieve the itching and fever, the Indians would often plunge into cold water, even in the winter.

---

**Buffalo Mortality for 1914** was 7,040 against 7,043 for 1913. The former represents a death rate of 15.5: 1000 population. 44 1/4% of the persons dying were born in Buffalo, which speaks well for the permanency of the population.

---

**Batavia** has taken preliminary steps toward a pure **Water Supply.**

---

**Centenarian.** Elijah Prentice Rogers of Batavia celebrated his 102d birthday January 8. He was born in Lyme, Conn.

---

**Medical Mayor.** Santa Monica, Cal., has adopted a charter providing that subsequent mayors shall be physicians of at least five years experience and that the mayor shall act as Health Commissioner. The election will be held in December.

---

**Andrea Vesalius** was born at Louvain, December 31, 1514. He was drowned in a ship-wreck off the island of Zante, on his return from a pilgrimage to the Holy Land, October 15, 1564. While mainly known as an anatomist, he was also a clinician. A painting by E. Slingeneyer in the Brassela museum is entitled "Vesale auscultant un malade" but apparently the physical examination is by percussion only. Owing to circumstances over which they had no control, the inhabitants were prevented from celebrating the quatercentenary of the birth of Vesalius, as they had expected.

---

**Typhoid in the U. S. Army.** The report for 1913, showed only 4 cases among 90,752 men. 2 cases had not been vaccinated, one had received only the initial injection and one had been inoculated four years previously.

---

**Hog Cholera** was discovered in a stye near Farnham, January 11.

---

**Tuberculosis Campaign.** Twenty million dollars were spent on organized attempts to control this disease, last year, in the U. S. Over a quarter was spent in N. Y. State and more than half in N. Y., Ill., Penn., Mass. and Col. We have been asked to add our voice to the appeal for more liberal support of this cause. We do so most heartily, yet with the qualification that no one form of relief should be emphasized to the exclusion of interest in others and that due consideration must be given to the reasonable limitation of enforced philanthropy which already makes up about a tenth of the entire tax rate—more than a quarter if public education is included. Buffalo Rochester and other localities in our territory have provided efficiently for the care of the tuberculous and few counties remain in the state which have not established special hospitals or which are very sparsely inhabited so that the problem is of minor importance numerically and in regard to the need of special institutions. Every little while, we receive mimeographed news notes, which do not contain news. The preparation and mailing of such matter means an expense which might well be applied to the direct care of the tuberculous and if publicity items were mailed to journals only when something of importance exists to be recorded, it would save a vast amount of time and insure greater interest. Mayor Fuhrmann has just urged, for Buffalo, a bond issue of $600,000, to erect on the West Farm site, a hospital for the care of advanced cases of tuberculosis.

---

**Regulation of Solicitation of Funds for Charity.** Sen. Geo. F. Thompson of Niagara Co. has introduced a bill requiring reports of receipts and expenditures by all charitable institutions soliciting money.

---

**Relaxation of Vaccination Requirements.** Assemblyman Tallett of Madison Co. has introduced an amendment to the public health laws. Vaccination is not to be absolutely required for admission to the public schools unless small pox actually exists in the school district or city. Vaccinations are not to be performed except by regularly licensed physicians and all virus must be approved by the state commissioner of health. We are rather inclined to favor the first provision of this bill—not because we do not believe in vaccination but because we do. A considerable minority of the population require fresh argument as to the value of vaccination, and, apparently, it can be presented to them only by allowing them to risk their convictions against the general verdict of the medical profession. We believe so strongly in the efficacy of vaccination that we hold that, with numerically insignificant exceptions, the vaccinated would be protected even in the presence of small pox without quarantine. It is, of course, best to avoid all possible chance of danger both by rigorous quarantine and by universal vaccination but there are many who cannot be convinced of danger till they have put their fingers on the buzz saw. We are almost ready to admit that the greatest good to the greatest number will be achieved by granting to those most obstreporous in their demands for their rights, the God-given liberty to find out whether the buzz saw will cut. A few fresh wounds will probably convince the majority that certain rules are posted for their own benefit and not for the personal benefit of the medical superintendents of the great mill in which we work.

---

**Snakes.** It sounds like cinematograph fiction of the wildest kind to speak of the danger of poisonous snakes in a thickly settled portion of a populous city, in a region long supposed to have been cleared of all except harmless reptiles but a snake with 8 rattles (crotalus adamantius, var. adamantius, Beauvois) as well as a bull snake (pityophis melanoleucus, Daudain) were recently killed in the basement of a church being wrecked at the corner of Utica and Main Street, Buffalo. Both are southern varieties and probably escaped from some traveling show. If the former had not been dormant on account of cold, it is highly probable that the boys who found it would have been bitten. Numerous cases of ophiopsia have been reported and while most of these are probably hysteric, the police believe that at least one other snake, probably a rattler, is at large. In spite of sceptic tendencies and the probability of the effect of cold weather, the possible danger somewhat offsets the hygienic benefit of leaving babies out doors for long periods unattended.

---

**Drugs Not Conforming to U. S. P. Standard.** The U. S. Hygienic Laboratory has examined 10,524 samples of 26 official articles and has rejected 31.2% of the samples, as not of full strength, adulterated or otherwise not fulfilling the requirements. The rejections varied from 7.5% for olive oil to 78.1% for asafoetida.

---

**Legislators, New York State.** As our readers frequently have occasion to write to legislators in regard to matters of public policy, we give a list of those representing the special field of this journal.

Senate: 38 Dist. J. Henry Walters, Rep., 312½ Genesee St., Syracuse; 39. Wm. H. Hill, Rep. Lestershire; 40. Chas. J. Hewitt, Rep., Locke; 41. Morris S. Halliday, Rep., Ithaca; 43. Chas. D. Newton, Rep., Geneseo; 44. Archie D. Sanders, Rep. Stafford; 45. George F. Argetsinger, Rep. Rochester; 46. John B. Mullan, Rep. Rochester; 47. George F. Thompson, Rep., Middleport; 48. Clinton T. Horton, Rep., Buffalo; 49. Samuel J. Ramsperger, Dem., Buffalo; 50. William P. Greiner, Dem., Buffalo; 51. George E. Spring, Rep., Franklinville.

Assembly: Cattaraugus, DeHart Ames, Rep., Franklinville; Cayuga, William Whitman, Rep., Venice; Chautauqua, A. Morell Cheney, Rep., Bemus Point, John Leo Sullivan, Rep., Dunkirk; Chemung, Horace C. Walker, Rep., Horseheads;

Chenango, Bert Lord, Rep., Afton; Erie, Allan Keeney, Rep., Buffalo, Ross Graves, Rep., Buffalo, Nicholas J. Miller, Rep., Buffalo, James M. Mead, Dem., Buffalo, Arthur C. McElroy, Dem., Buffalo, Peter C. Jerzewski, Rep., Buffalo, John F. Heim, Rep., Lancaster, Leonard W. Gibbs, Rep., Buffalo, Frank B. Thorn, Rep., Buffalo; Genesee, Louis H. Wells, Rep., Pavilion; Herkimer, Seldon C. Clobridge, Rep., Herkimer; Jefferson, Willard S. Augsbury, Rep., Antwerp; Madison, Morrell E. Tallett, Rep., DeRuyter; Monroe, James A. Harris, Rep., Penfield, Simon L. Adler, Rep., Rochester, John R. Powers, Rep., Rochester, Frank Dobson, Rep., Charlotte, Franklin W. Judson, Rep., Gates; Niagara, William Bewley, Rep., Lockport, Alan N. Parker, Rep., Niagara Falls; Oneida, Fred S. Emden, Dem., Utica, Chas. J. Fuess, Rep., Utica, J. Brayton Fuller, Oldfield, Rep., Bath, Richard M. Prangem, Rep., Hornell; Tioga, Wilson S. Moore, Rep., Candor; Tompkins, John W. Preswick, Rep., Ithaca; Wayne, Riley A. Wilson, Rep., Savannah; Wyoming, John Knight, Rep., Arcade; Yates, Edwin C. Rep., Marcy; Onondaga, Edward Arnts, Rep., Syracuse; J. Leslie Kincaid, Rep., Syracuse, Jacob R. Buechler, Rep., Syracuse; Ontario, Heber E. Wheeler, Rep., East Bloomfield; Seneca, William J. Maier, Rep., Seneca Falls; Steuben, Reuben B. Gillette, Rep., Penn Yan.

---

**Whisker Legislation.** A bill has been introduced in Massachusetts, prohibiting anyone who performs or assists at an operation, from wearing beard or mustach. Ridiculous but quite as defensible as most of the modern type of reform legislation.

---

**Public Health Service Examinations** for assistant surgeons will be held at various places March 8. We have frequently repeated the requirements and mentioned the advantages of this service for medical men. Candidates should apply immediately to the Surgeon General at Washington, for detailed information.

---

**Elmira** has joined the progressives by opening a "Tuberculosis Dispensary."

---

**Ambulance Construction Commission. Prize Competition.** This is the first great war in which field motor ambulances have been used, so that opportunities for improvement exist. Mr Henry S. Wellcome has, therefore, secured this commission which includes Sir Frederick Treves, representing the British Red Cross Society, Sir Claude Macdonald and Sir John Furley of the St. John Ambulance Association, and representatives of the army and navy. For obvious reasons, the Commission will limit its consideration to bodies, for chassis of sizes to be designated. It offers in competition, for designs and specifications submitted before June 30, prizes of 1,000, 500 and 300 pounds, respectively. For details, apply to the Secretary of the Commission, 10 Henrietta St., Cavendish Square, London.

---

**Examination of Dentists for the U. S. Army.** The Surgeon-General of the Army announces that examinations for the appointment of Acting Dental Surgeons will be held at Fort Slocum, New York; Columbus Barracks, Ohio; Jefferson Barracks, Missouri; Fort Logan, Colorado, and Fort McDowell, California, on Monday, April 12, 1915.

Application blanks and full information concerning these examinations can be procured by addressing the "Surgeon-General, U. S. Army, Washington, D. C."

The essential requirements to securing an invitation are that the applicant shall be a citizen of the United States, shall be between 21 and 27 years of age, a graduate of a dental school legally authorized to confer the degree of D. D. S., and shall be of good moral character and habits.

Acting Dental Surgeons are employed under a three years' contract, at the rate of $150.00 per month. They are entitled to traveling allowances in obeying their first orders, in changing stations, and in returning to their homes at termination of service. They also have a privilege of purchasing certain supplies at the Army commissary. After three years service if found qualified, they are promoted to the grade of dental surgeon with the rank of first lieutenant, and receive thereafter the pay and allowances appertaining to that rank.

In order to perfect all necessary arrangements for the examination, applications must be in the possession of the Surgeon-General at least two weeks before the date of examination. Early attention is therefore enjoined upon all intending applicants. There will be 9 vacancies to be filled.

---

**The Women's Educational and Industrial Union of Buffalo** has tendered its building on Niagara Square for the College Department of the University of Buffalo, providing $100,000 can be raised. The Union will not go out of existence but will continue to exercise its general purpose along new lines. In fact the Union owes whatever is the corresponding feminine form of virility, to its policy not to ride a hobby but to change the details of its work so as to do something needed and not to conflict with or duplicate the work of other organizations. The editor has followed the work of the Union for many years since its inception by a group of women who included many of his most intimate personal friends and some of his family. He had the honor of becoming its first male member. If all philanthropic institutions had so firm a grasp of the principle of elasticity to provide for practically necessary work, there would not be the complaint as to the relative inefficiency of charity.

---

**Drug Patents.** Congressman Calvin D. Paige of Massachusetts has introduced the following amendment: "No patent shall be granted upon any drug, medicine, medicinal chemical, coal-tar dyes or colors, or dyes obtained from alizarin, anthracene, carbazol, and indigo, except insofar as the same relates to a definite process for the preparation of said drug, medicine, medicinal chemical, coal-tar dyes or colors, or dyes obtained from alizarin, anthracene, carbazol, or indigo."

---

**Ancient Intravenous Medication.** Michel Etmuller, in 1691, credits this method to the English and probably to Voren of Oxford. He cites a case at Dantzig in which a syphilitic soldier was given an intravenous injection of scammonium and guaiacum, with healing of intractable ulcers in 3 days. (La Chronique Med.) Possibly it is not so much the specific action of a given drug as the fact of its prompt and massive effect when given intravenously that gives the impression of superiority of recent over older preparations.

---

**Bacteriologic Standards for Meat.** E. B. Newton, "Am. Jour. Public Health," May 1914, states that one million bacteria per gram is taken as the limit at which putrefaction may be considered to begin. 50% samples of Hamburg steak exceeded ten million, which he proposes as a fair standard of this form of meat.

